# "Look What the Cat Dragged In": Delayed-Onset Hypersensitivity Pneumonitis Due to Pregnancy

**DOI:** 10.7759/cureus.97031

**Published:** 2025-11-17

**Authors:** Robin Roberts, Angharad Rowles, Hana Alachkar, Claire Kilduff

**Affiliations:** 1 Medicine, Ysbyty Gwynedd Hospital, Bangor, GBR; 2 Immunology, Royal Liverpool University Hospital, Liverpool, GBR; 3 Respiratory Medicine, Ysbyty Gwynedd Hospital, Bangor, GBR

**Keywords:** avian protein, bird fancier's lung, environmental allergen, hypersensitivity pneumonitis, multiple lung nodules, peripartum physiological changes, pulmonary immunology

## Abstract

Hypersensitivity pneumonitis (HP) is an immune-mediated inflammatory lung disease triggered by the inhalation of environmental antigens. Bird fancier's lung is a common subtype, whereby the hypersensitivity reaction is triggered by exposure to avian proteins from bird feathers and droppings. This case describes a woman in her 20s, presenting at 16 weeks postpartum, with progressive dyspnoea, dry cough, and hypoxia.

A thorough social history revealed significant exposure to a pigeon after the woman had rescued a pigeon from her cat and nursed it back to health during her third trimester. Computed tomography (CT) imaging revealed diffuse pulmonary nodules and air trapping, and subsequent serology confirmed elevated IgG to avian proteins. The patient was diagnosed with bird fancier's lung and managed with corticosteroids. The pigeon was relocated to avoid further exposure.

It is proposed that the Th2-dominant immune profile during pregnancy leads to an anti-inflammatory state, suppressing Th1-driven conditions such as HP and delaying symptom onset until the postpartum immune rebound. Recognition of this immunological shift is critical in postpartum patients with atypical respiratory symptoms. Timely history-taking, specialist input, and consideration of environmental triggers were key to swift diagnosis and management in this case.

## Introduction

Hypersensitivity pneumonitis (HP), previously referred to as "extrinsic allergic alveolitis", is a collection of immune-mediated, inflammatory lung diseases [1]. The disease is triggered by the inhalation of environmental allergens in susceptible individuals [2]. Most commonly, these are organic antigens, from exposure to certain bacteria, fungi, plant proteins, and animal proteins [3]. Exposures classically occur in certain hobbies or occupations and are often classified as such, e.g., farmer's lung, hot tub lung, and cheese worker's lung [3]. Amongst these subtypes is bird fancier's lung, whereby exposure to avian proteins from feathers and bird droppings triggers the hypersensitivity reaction [4].

Allergenic exposure activates both the innate and adaptive immune systems, releasing cytokines and chemokines [5]. This leads to both type III and type IV hypersensitivity reactions mediated by immune complexes and Th1 cells, with subsequent granuloma formation [6]. While acute HP can present within hours to days of exposure, subacute and chronic forms may develop over weeks to years [3]. Clinical manifestations include dyspnoea, dry cough, chest tightness, and associated flu-like symptoms: fevers, chills, headache, anorexia, and fatigue [7].

The American Thoracic Society has established a diagnostic algorithm that incorporates both investigation results and multidisciplinary team discussion, highlighting the importance of a detailed social history to identify potential allergenic exposures [7]. Investigations include high-resolution computed tomography (CT), serum antibodies against offending antigens, bronchoalveolar lavage, and, in rare cases, lung biopsy [8]. The mainstay of management involves removing exposure to the triggering allergen, along with immunosuppression, generally corticosteroids as first-line [9].

## Case presentation

History

A woman in her 20s was referred to the Medical Assessment Unit with a one-month history of progressively worsening dyspnoea, dry cough, and coryza. She had no significant past medical history, but was 16 weeks postpartum, following an uncomplicated vaginal delivery at term.

She initially presented to her general practitioner (GP) at 13 weeks postpartum, with a one-week history of sore throat, coryza, non-productive cough, and shortness of breath. At this time, her symptoms were mild, and her presentation was attributed to a likely viral upper respiratory tract infection. She re-presented to her GP in week 2 of illness and was trialled on a beclometasone and formoterol (Fostair) inhaler and a proton pump inhibitor, under the working diagnosis of possible asthma or gastro-oesophageal reflux.

By the third presentation, she was four weeks into the illness, and her dyspnoea had significantly worsened. She reported significant shortness of breath on mild exertion, such as walking up one flight of stairs, putting out the washing, or carrying her baby between rooms. She had also developed chest discomfort and tightness, along with subjective fevers and cold sweats. She described feeling lightheaded at times and experienced nausea after sudden outbursts of coughing. Concerned about the possibility of a pulmonary embolism, the GP referred her to secondary care for further assessment.

The patient did not experience any orthopnoea, peripheral oedema, haemoptysis, or weight loss. She has no known allergies and no history of asthma or atopy. Her only regular medication was the desogestrel contraceptive pill, which she had commenced three weeks prior. Her only relevant family history was chronic obstructive pulmonary disease (COPD) in her mother, who was an ex-smoker.

A detailed social history was obtained, which revealed there were no smokers or vapers in her household, nor did she take any illicit substances. She had no recent foreign travel history and no exposure to infectious contacts. She worked part-time managing and cleaning well-kept holiday homes, hence had minimal exposure to mould. For the past three years, she has resided on a smallholding with her partner. She kept several animals, including goats, horses, dogs, cats, and a parakeet.

Notably, at approximately 28 weeks of gestation, her pet cat caught a pigeon and brought it into the family home. The patient and her partner rescued the pigeon and nursed it back to health. For many months, the pigeon returned every night, flying in through her bedroom window and sleeping on top of the parakeet's cage. At times, the pigeon would fly around the bedroom, causing bloom and dropping to disperse around the room.

Examination findings included the following: a sinus tachycardia of 115 bpm, but a strong, regular radial pulse, and normal heart sounds. Chest auscultation revealed bi-basal crepitations, and she was hypoxic at 89% on air, so she was commenced on 2L oxygen via nasal cannula. Her calves were soft and non-tender, and there were no signs of a deep-vein thrombosis (DVT). Her jugular venous pressure (JVP) was not elevated, and she was clinically euvolemic. There was no evidence of myalgia, ear, nose, and throat (ENT) symptoms, ulcers, rash, abdominal discomfort, or meningism.

Investigations

Routine admission bloods, including a D-dimer assay, were obtained on arrival to the hospital (see Table 1). Inflammatory markers were mildly elevated (C-reactive protein (CRP): 33 mg/L; white cell count (WCC): 12.9×10⁹/L), with a corresponding mild neutrophilia. Additional initial laboratory bloods revealed normal troponin and N-terminal pro-B-type natriuretic peptide (NT-proBNP) levels. Her electrocardiogram (ECG) showed sinus tachycardia without further abnormalities.

**Table 1:** Laboratory results CRP: C-reactive protein; NT-proBNP: N-terminal pro-B-type natriuretic peptide

Test	Value	Normal range
White cell count	12.9×10^9^/L	4-11×10^9^/L
Platelet count	418×10^9^/L	150-400×10^9^/L
Neutrophil count	10.3×10^9^/L	1.7-7.5×10^9^/L
Lymphocyte count	1.3×10^9^/L	1-4.5×10^9^/L
Eosinophil count	0.2×10^9^/L	0-0.4×10^9^/L
CRP	33 mg/L	<5 mg/L
D-dimer	351 ng/mL	<500 ng/mL
Fibrinogen	4.6 ­g/L	2-4 g/L
Troponin	<3 ng/L	<14 ng/L
NT-proBNP	17 pg/mL	<100 pg/mL
Total IgE	446 ­kU/L	0-87 kU/L
IgG to budgerigar (parakeet)	115 mg/L	<20 mg/L
IgG to pigeon	>200 mg/L	<20 mg/L
IgG to parrot	34.1 ­mg/L	<20 mg/L
IgG to *Micropolyspora faeni* (farmer's lung precipitant)	10.4 mg/L	<20 mg/L
IgG to *Aspergillus fumigatus*	12 mgA/L	<25 mgA/L

**Figure 1:**
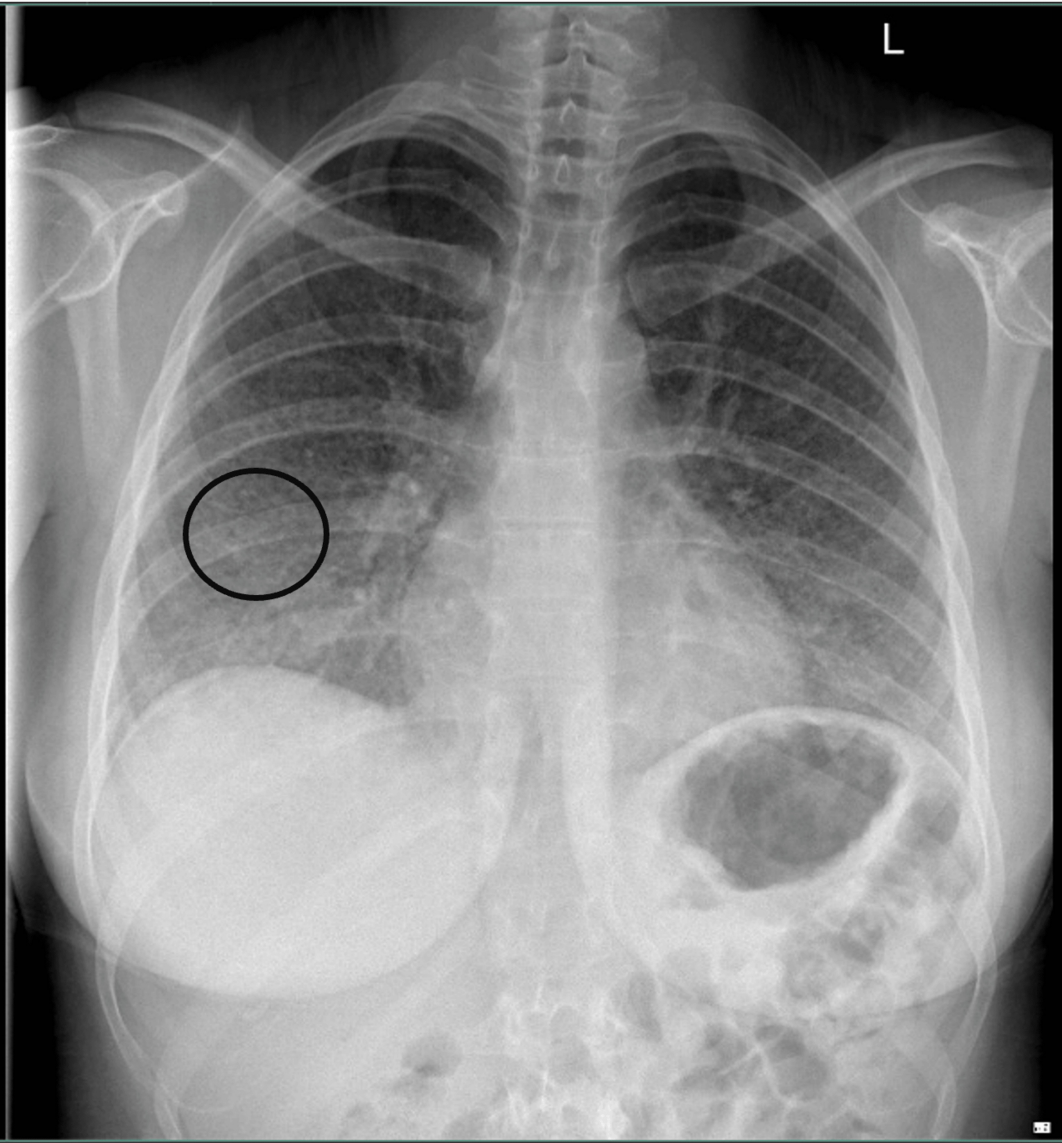
Admission chest X-ray. Nodular soft tissue pattern throughout both lung fields (best appreciated within circled area). No focal consolidation. Heart and mediastinal contours normal

**Figure 2:**
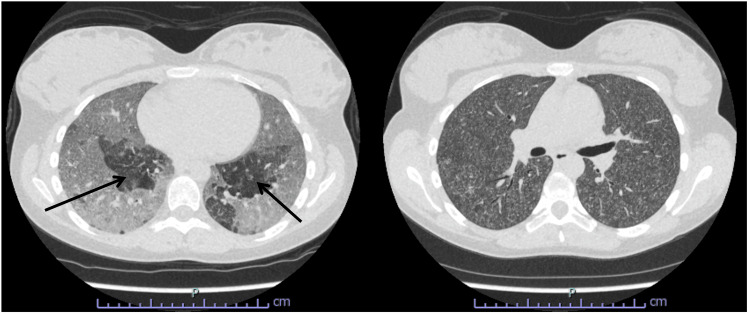
High-resolution CT of the thorax. Diffusely abnormal lungs bilaterally, with innumerable soft tissue centrilobular nodules of varying sizes, most of which are fluffy and ill-defined. In the lower lobes, there are areas of air trapping (see arrows). No mediastinal or hilar adenopathy CT: computed tomography

A plain chest radiograph revealed widespread parenchymal changes, notably multiple nodular lesions (Figure 1). A respiratory consult was sought, and a full viral panel, along with high-resolution CT of the chest, was requested. The high-resolution CT of the chest identified numerous nodular lesions distributed throughout both lungs, along with air trapping in the lower lobes (Figure 2). Following discharge, serological testing returned positive IgG reactivity to pigeon, budgerigar (parakeet), and parrot avian proteins (Table 1). The vasculitis screen, extended viral respiratory panel, and antibody profiles for myositis and scleroderma all returned negative.

Differential diagnoses

HP: Bird Fancier's Lung

The patient had a clear history of a new allergenic stimulus (pigeon) introduced to the family home. Her symptoms of progressive dyspnoea and dry cough were consistent with interstitial lung disease. Imaging findings of innumerable nodules and air trapping supported this diagnosis. Immunoglobulins strongly positive for avian precipitins later supported the diagnosis: IgG to pigeon >200 mg/L, IgG to budgerigar (parakeet) 115 mg/L, and IgG to parrot 34.1 mg/L (normal <20 mg/L).

Infection

A history of cough, dyspnoea, subjective fevers, and elevated inflammatory markers raises the suspicion of a bacterial or viral pneumonia. Given the initial coryzal symptoms, the history is in keeping with an initial viral respiratory tract infection, which may have progressed to a secondary bacterial pneumonia. However, the presence of bilateral nodular findings in an immunocompetent patient, coupled with the known new environmental exposure to avian allergens, suggests that an inflammatory aetiology is more likely.

Pulmonary Embolism

The patient's significant dyspnoea, hypoxia, pleuritic chest discomfort, and tachycardia all raised suspicion of a pulmonary embolism. This was especially concerning given our patient was in the postpartum period, a well-established risk factor for DVT and pulmonary embolism. Reassuringly, her D-dimer level was negative, essentially excluding the diagnosis. Her imaging findings further supported an inflammatory pathology over a pulmonary embolism.

Malignancy

Diffuse nodular findings on the plain radiograph raised suspicion of malignancy; however, this would be unusual in a patient so young, with no smoking history. Her symptoms were also relatively acute in onset and more in keeping with an inflammatory or infective aetiology. Further characterization of these nodules on high-resolution CT, along with the associated air trapping and absence of lymphadenopathy or masses, made malignancy even less likely.

Treatment

Initial management included oral amoxicillin and clarithromycin to cover for potential infection, of which she completed a five-day course. Supplemental oxygen was administered to maintain target saturations above 94%, with a maximum requirement of 3 L/minute, delivered via nasal cannulae.

The history of new allergenic exposure, along with subsequent imaging findings, was highly suggestive of HP. The patient was therefore commenced on high-dose (60 mg) oral prednisolone once daily, along with lansoprazole for gastric protection and Adcal D3 for bone protection. The patient and her partner were counselled regarding the likely diagnosis of bird fancier's lung. They subsequently relocated both the pigeon and budgie and deep-cleaned the house to avoid further antigen exposure. 

Outcome and follow-up

The patient required a six-day admission to the hospital, where she received high-dose oral prednisolone, in combination with empirical oral antibiotics. After five days of treatment, she was successfully weaned off oxygen and discharged home. She was advised to reduce her prednisolone dose by 5 mg/week until completed.

**Figure 3:**
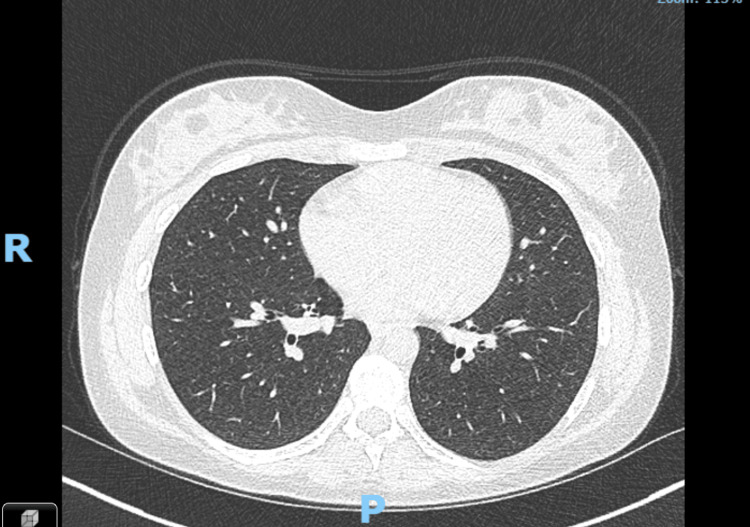
Repeat high-resolution CT of the thorax: there is complete resolution of previously noted extensive ill-defined centrilobular nodularity with significant improvement in the mosaic attenuation pattern. There are persistent areas of subtle low density in the lower lobes corresponding to previous areas of air trapping. No concerning focal lung lesion or consolidation CT: computed tomography

During a follow-up telephone call in the respiratory outpatient clinic, the patient reported her symptoms had completely resolved. She no longer has any limitations on her daily activities and feels back to her normal self. She underwent a follow-up high-resolution CT of the thorax to ensure the resolution of the pulmonary nodules and air trapping (Figure 3). 

## Discussion

Exposure to bird-derived antigens, commonly found in droppings and feathers, elicits both humoral and cellular immune responses. Innate immune cells present antigens to T cells, simultaneously promoting cytokine secretion (e.g., IL-12, IFN-γ) and driving Th1 polarization [10] while also activating B lymphocytes to produce immunoglobulins [5,7]. These antibodies bind to antigens, forming immune complexes that activate the classical complement pathway and induce tissue injury [11].

During pregnancy, the maternal immune system undergoes profound modulation. T-helper (CD4⁺) cells decline during the second and third trimesters, and T-cytotoxic (CD8⁺) cells decrease in the third trimester [12]. Both populations typically return to baseline by around four months postpartum [13].

A key feature of pregnancy is a shift from Th1 to Th2 dominance, favouring an anti-inflammatory immune profile. This shift is associated with the reduced production of Th1 cytokines such as IL-2 and IFN-γ [14] and increased levels of Th2 cytokines including IL-4 and IL-10 [15]. Progesterone contributes to this transition by suppressing Th1 activity [16] and promoting Th2 cytokines like IL-4 and IL-5 [17]. Clinically, Th1- and Th17-mediated autoimmune diseases often improve during pregnancy [18-20], while Th2-type conditions may worsen [21]. This Th2 bias initiates early in gestation [22,23] and typically resolves by four weeks postpartum [24].

Additionally, the number of regulatory T cells (Tregs), key immunosuppressive cells essential for maintaining foetal tolerance, increases significantly during pregnancy, peaking in the second trimester [25] and sometimes remaining elevated postpartum. Tregs mediate peripheral tolerance by suppressing CD4⁺ and CD8⁺ T-cell proliferation and cytokine release, inhibiting B-cell antibody production, reducing NK cell cytotoxicity, and modulating dendritic cell activity.

These immune adaptations protect the mother and foetus from pathogens while minimizing harmful immune responses against the semi-allogeneic foetus. Although pregnancy is not a state of global immunosuppression, these changes result in altered susceptibility to specific infections and immune-mediated conditions. So, the maternal immune system during pregnancy shifts toward a Th2-dominant, anti-inflammatory profile, particularly in the second and third trimesters. This state, marked by reduced Th1 cytokines, dampened CD4⁺ T-cell activity, and elevated Tregs, suppresses the Th1-mediated immune responses involved in bird fancier's lung. As bird fancier's lung is Th1-driven, the immunological shift likely delayed disease onset despite continued avian antigen exposure in our patient.

Following delivery, the immune system progressively reverts back to a Th1-prone state, restoring pro-inflammatory responses. Consequently, immune recognition and response to bird antigens may only become clinically apparent postpartum, resulting in the development of acute bird fancier's lung months after initial exposure began.

Simply put, we propose that our patient was in an anti-inflammatory state while pregnant, hence did not develop clinical manifestations of bird fancier's lung until her immune system returned to its usual inflammatory state many months later, at three months post-partum.

## Conclusions

This case highlights the complex changes in immune physiology during pregnancy and how this might influence the pathophysiology of HP. During pregnancy, the immune system adopts a Th2-dominant, anti-inflammatory profile characterized by reduced Th1 cytokines, dampened CD4⁺ T-cell activity, and elevated levels of Tregs. This immunological state likely suppresses the Th1-mediated immune responses involved in bird fancier's lung. This potentially led to delayed disease onset in our patient, despite ongoing exposure to avian antigens throughout late pregnancy.

Following delivery, the postpartum immune rebound towards a Th1-prone, pro-inflammatory state may have unmasked the hypersensitivity reaction, leading to symptom onset several months after exposure began. This case, therefore, underscores the importance of recognizing pregnancy and the postpartum period as unique immunological windows, during which immune-mediated diseases may present atypically or with delayed onset. Comprehensive history-taking, including environmental and animal exposure, combined with early multidisciplinary input, remains essential for the timely diagnosis and management of rare conditions such as bird fancier's lung in postpartum patients.
